# Sex-Differences in Response to Treatment with Liraglutide 3.0 mg

**DOI:** 10.3390/jcm13123369

**Published:** 2024-06-07

**Authors:** Ilaria Milani, Gloria Guarisco, Marianna Chinucci, Chiara Gaita, Frida Leonetti, Danila Capoccia

**Affiliations:** Department of Medico-Surgical Sciences and Biotechnologies, Faculty of Pharmacy and Medicine, University of Rome La Sapienza, 04100 Latina, Italy; ilaria.milani@uniroma1.it (I.M.); gloria.guarisco@gmail.com (G.G.); chinuccimarianna@gmail.com (M.C.); chiara.gt17@gmail.com (C.G.); frida.leonetti@uniroma1.it (F.L.)

**Keywords:** obesity, sex, GLP1-RAs, liraglutide, weight loss

## Abstract

**Background**: Sex differences characterize the prevalence and attitudes toward weight management. Despite limited evidence suggesting greater weight loss in women with anti-obesity pharmacotherapy, sex-specific analysis remains underexplored. This retrospective study aimed to evaluate the sex-specific response to liraglutide 3.0 mg treatment in people with obesity without type 2 diabetes (T2D). **Methods**: Data were collected from 47 patients (31 women, 16 men) with age > 18 years; BMI ≥ 30 kg/m^2^; absence of T2D; and exclusion of prior anti-obesity treatment, comorbidities, or bariatric surgery. Only patients who maintained the liraglutide 3.0 mg dose for at least 6 months were included. **Results**: Both sexes showed significant reductions in weight and BMI at 3 and 6 months. Men achieved greater weight loss (WL), BMI reduction, %WL, WL > 5%, and >10% than women, and they also showed more significant improvements in metabolic parameters (total and LDL cholesterol, Fibrosis-4 Index FIB-4). No significant sex differences were observed in glucose metabolism or renal function. **Conclusions**: This study showed a greater therapeutic effect of liraglutide 3.0 mg in men. Given men’s higher risk of cardiovascular disease (CVD), and underrepresentation in clinical weight loss programs, these findings may increase male engagement and improve their CVD risk.

## 1. Introduction

Over the past 2 decades, the increase in morbidity and mortality associated with excess body weight has made obesity a major health problem, with implications for both public health and healthcare expenditure [1,2].

Results from studies analyzing changes in obesity rates from 1980 to 2019 have shown a significant increase in prevalence in both men (from 3.2% to 12.2%) and women (from 6% to 15.7%). In particular, in recent years, young men aged 20–44 years appear to have a higher prevalence of overweight/obesity than young women, but this trend seems to be the reverse after the age of 45, probably due to menopausal changes in women [3]. In Italy, excess body weight was found to be higher in men than in women (53.6% vs. 35.7%), with an increasing trend with age [4].

In general, women are more likely to be diagnosed with obesity and to seek and receive all types of obesity treatment, including lifestyle modification, pharmacotherapy, and bariatric surgery. This propensity for intervention among women raises concerns about sex/gender disparities in obesity treatment [5].

Several studies have shown that weight loss significantly reduces the risk of obesity-related complications [1]. In addition, the availability of new anti-obesity drugs has renewed interest in pharmacological therapy of obesity, which has been recognized as an innovative strategy for its management [6]. However, the clinical approach to obesity often does not take into account sex/gender differences, which can influence the management of obesity and improve the treatment of obesity [5].

Orlistat, naltrexone/bupropion, and liraglutide are approved anti-obesity drugs in Italy. Among the Glucagon-like peptide-1 receptor agonists (GLP1-RAs), liraglutide has been shown to promote weight loss in patients with T2D [7], leading to its approval for chronic weight control at a daily dose of 3.0 mg in people who are overweight and without T2D [8]. Despite previous clinical trials demonstrating the superior efficacy of liraglutide 3.0 mg in promoting weight loss in women compared with men [9], there has been limited real-world research on the sex-specific clinical response to this drug. 

Since pharmacodynamic and pharmacokinetic analyses suggest that sex may significantly influence the effect of these drugs [7], we decided to plan this retrospective study to evaluate a potential sex difference in response to liraglutide 3.0 mg treatment in a cohort of people with obesity and without T2D.

## 2. Materials and Methods

### 2.1. Study Population

We conducted a single-center retrospective study of patients with obesity and without T2D treated with liraglutide 3.0 mg who attended the Obesity Unit of the Santa Maria Goretti Hospital in Latina, Italy, from October 2022 to October 2023, according to the following criteria:

1. inclusion criteria: age > 18 years, presence of obesity (body mass index BMI ≥ 30 kg/m^2^), absence of T2D according to ADA criteria [10], and other metabolic comorbidities and treatments;
2. exclusion criteria: previous anti-obesity treatment (GLP1-RAs or others) and/or bariatric surgery, pregnancy.

Liraglutide treatment was titrated from 0.6 mg to 3.0 mg weekly. Only patients who reached the maximum dose of liraglutide (3.0 mg) during the 5-week titration period, and maintained this dose for at least 6 months of follow-up, were included in the study. In our obesity unit, patients with obesity generally undergo nursing counseling, both regarding healthy lifestyle and drug titration.

### 2.2. Data Collection and Definition

Demographic and anthropometric parameters such as age, weight and BMI, and clinical data including glucose metabolism, lipid profile, liver, and renal function were collected retrospectively from the electronic reporting system of our obesity unit. 

Body weight (BW), height, and BMI (calculated as weight (kg) divided by the square of height (m)) were used to express anthropometric parameters. Measurement of fasting blood glucose and glycated hemoglobin (FBG and HbA1c) allowed evaluation of glucose metabolism. The lipid profile was assessed by total cholesterol, LDL cholesterol, HDL cholesterol, and triglycerides (TGs). To examine liver function, transaminases (AST and ALT) were measured, and the Fibrosis-4 Index score (FIB-4) was calculated using the following formula: (age [years] × aspartate transaminase AST [U/L])/(platelets [109/L] × (alanine transaminase ALT [U/L])1/2) [11]. Renal function was assessed by serum creatinine.

The anthropometric data were collected at baseline T0 and after 3 months T1 (2–4 months, average 3 months) and 6 months T2 (5–7 months, average 6 months) of treatment. The metabolic parameters were collected at baseline (T0) and after 6 months of treatment (T2). 

At each visit, medical staff recorded the patient’s weight and defined weight loss as the difference between the follow-up weight and the baseline weight. BMI change was also calculated as the difference between the follow-up BMI and the baseline BMI. If there were multiple visits within 2–4 months, the lowest weight was used, and if there were multiple visits within 5–7 months of the last visit, the weight closest to 6 months was used.

The percentage weight loss (%WL), and the percentage of patients who lost at least 5% or 10% of their initial weight at 3 and 6 months, respectively, were recorded.

The prescription of anti-obesity pharmacotherapy is not reimbursed by the Italian healthcare system, and the high cost represents one of the main barriers to treatment, as not all patients are able to afford it. In some real-world studies, patient payment for obesity medications has been identified as a cause of higher discontinuation rates [12]. In this study, during data collection, the patient’s visit date, prescribed dose, number of liraglutide prescriptions, any adverse effects, and whether these effects led to discontinuation of the medication were reviewed to determine whether the medication had been discontinued. This approach has the potential to provide adequate data for the indirect assessment of patient adherence.

### 2.3. Statistical Analysis

A total of 47 participants living with obesity were required for this study given a power analysis assuming an alpha (α) value of 0.05, a power level of 0.80, and large effect size relating to a Cohen’s d of 0.85. The power estimation was performed with software G power version 3.1.9.7. 

Means (±standard deviation) were used to represent baseline variables. Percentages were used for categorical variables. Comparisons between groups were performed using the independent *t*-test. A *p* < 0.05 (5%) was considered significant in all statistical analyses performed with IBM SPSS Statistics software for Windows, version 21.0.

## 3. Results

Initially, we reviewed the medical records of 72 patients with obesity who started treatment with liraglutide. Of these, 47 patients who reached the maximum dose of liraglutide (3.0 mg) during the 5-week titration period and maintained this dose for at least 6 months of follow-up were included in the study. 

Data were collected from 31 (65.9%) women and 16 (34.0%) men with a mean age of 50.8 (±11.8) years, body weight of 110.2 (±14.1) kg, and BMI of 39.3 (±5.4) kg/m^2^. 

The baseline analysis (T0) showed no significant differences in BMI (37.5 ± 5.6 kg/m^2^ vs. 40.1 ± 5.2 kg/m^2^, *p* = 0.12) and weight (115.4 ± 18.1 kg/m^2^ vs. 108.3 ± 12.2 kg/m^2^, *p* = 0.10) between men and women (Table 1). 

In the male group, the results showed a significant reduction in BW and BMI at 3 months (−10.7 ± 6.1 kg; −3.6 ± 2.4 kg/m^2^, *p* < 0.0001) and at 6 months (−17.9 ± 6.7 kg; −6.0 ± 2.7 kg/m^2^, *p* < 0.0001). Analysis of metabolic parameters showed a significant reduction in LDL cholesterol (−19.0 ± 16.5, *p* < 0.0001) mg/dL and FIB-4 (−0.25 ± 0.23, *p* = 0.05). No significant differences were observed in the change in HbA1c (−0.23 ± 0.9%, *p* = 0.57), FBG (−8.1 ± 16.4 mg/dL, *p* = 0.23), total cholesterol (−14.0 ± 32.6 mg/dL, *p* = 0.23), HDL cholesterol (−2.0 ± 14.5 mg/dL, *p* = 0.70), TG (−9.1 ± 59.9, *p* = 0.68), creatinine (−0.02 ± 0.14 mg/dL, *p* = 0.67), AST (−3.5 ± 5.6 U/L, *p* = 0.12), and ALT (−5.0 ± 15.1 U/L, *p* = 0.38). In the female group, the results showed a significant reduction in body weight and BMI at 3 months (−7.1 ± 3.1 kg; −2.6 ± 1.1 kg/m^2^, *p* < 0.0001) and at 6 months (−11.9 ± 5.3; −4.4 ± 1.9 kg/m^2^, *p* < 0.0001). Analysis of metabolic parameters showed a significant reduction in FBG (−5.4 ± 9.4 mg/dL, *p* = 0.03). No significant differences were found in HbA1c (−0.16 ± 0.6%, *p* = 0.31), total cholesterol (9.5 ± 22.1 mg/dL, *p* = 0.11), LDL cholesterol (6.8 ± 21.2 mg/dL, *p* = 0.20), HDL cholesterol (1.1 ± 7.1 mg/dL, *p* = 0.59), TG (2.8 ± 51.9, *p* = 0.83), creatinine (−0.02 ± 0.06 mg/dL, *p* = 0.28), AST (−2.1 ± 4.8 U/L, *p* = 0.10), ALT (−3.4 ± 7.7 U/L, *p* = 0.10), and FIB-4 (−0.003 ± 0.12, *p* = 0.92). 

In the male group, the results showed a significant reduction in BW and BMI at 3 months (−10.7 ± 6.1 kg; −3.6 ± 2.4 kg/m^2^, *p* < 0.0001) and at 6 months (−17.9 ± 6.7 kg; −6.0 ± 2.7 kg/m^2^, *p* < 0.0001). Analysis of metabolic parameters showed a significant reduction in LDL cholesterol (−19.0 ± 16.5, *p* < 0.0001) mg/dL and FIB-4 (−0.25 ± 0.23, *p* = 0.05). No significant differences were observed in the change in HbA1c (−0.23 ± 0.9%, *p* = 0.57), FBG (−8.1 ± 16.4 mg/dL, *p* = 0.23), total cholesterol (−14.0 ± 32.6 mg/dL, *p* = 0.23), HDL cholesterol (−2.0 ± 14.5 mg/dL, *p* = 0.70), TG (−9.1 ± 59.9, *p* = 0.68), creatinine (−0.02 ± 0.14 mg/dL, *p* = 0.67), AST (−3.5 ± 5.6 U/L, *p* = 0.12), and ALT (−5.0 ± 15.1 U/L, *p* = 0.38). In the female group, the results showed a significant reduction in body weight and BMI at 3 months (−7.1 ± 3.1 kg; −2.6 ± 1.1 kg/m^2^, *p* < 0.0001) and at 6 months (−11.9 ± 5.3; −4.4 ± 1.9 kg/m^2^, *p* < 0.0001). Analysis of metabolic parameters showed a significant reduction in FBG (−5.4 ± 9.4 mg/dL, *p* = 0.03). No significant differences were found in HbA1c (−0.16 ± 0.6%, *p* = 0.31), total cholesterol (9.5 ± 22.1 mg/dL, *p* = 0.11), LDL cholesterol (6.8 ± 21.2 mg/dL, *p* = 0.20), HDL cholesterol (1.1 ± 7.1 mg/dL, *p* = 0.59), TG (2.8 ± 51.9, *p* = 0.83), creatinine (−0.02 ± 0.06 mg/dL, *p* = 0.28), AST (−2.1 ± 4.8 U/L, *p* = 0.10), ALT (−3.4 ± 7.7 U/L, *p* = 0.10), and FIB-4 (−0.003 ± 0.12, *p* = 0.92). 

When comparing the two groups (men vs. women), men showed more significant reductions in weight (−10.7 ± 6.1 kg vs. −7.1 ± 3.1 kg, *p* < 0.0001) at 3 months and at 6 months (−17.9 ± 6.7 kg vs. −11.9 ± 5.3 kg, *p* < 0.0001) of follow-up than women (Table 2).

Similar results were found for BMI, with men showing higher reduction (−3.6 ± 2.4 kg/m^2^ vs. −2.6 ± 1.1 kg/m^2^, *p* = 0.08) at 3 months and at 6 months (−6.0 ± 2.7 kg/m^2^ vs. −4.4 ± 1.9 kg/m^2^, *p* = 0.07) of follow-up than women (Table 2). 

At 3 months, WL > 5% was achieved in 93.7% (N = 15/16) of men vs. 58.0% (N = 18/31) of women (*p* < 0.0001), and %WL was higher in men than in women (−9.2 ± 5.1% vs. −6.5 ± 2.9%, *p* < 0.0001) (Table 2); at 6 months, WL > 10% was observed in 87.5% (N = 14/16) of men vs. 29.0% (N = 9/31) of women (*p* < 0.0001), and %WL was higher in men than women (−15.2 ± 5.4% vs. −10.5 ± 4.4%, *p* < 0.0001) (Table 2).

No significant differences in metabolic parameters were observed between the two groups, except for a significantly greater reduction in total cholesterol (−14.0 ± 32.6 mg/dL vs. 9.5 ± 22.1 mg/dL, *p* < 0.0001) and LDL cholesterol (−19.0 ± 16.5 mg/dL vs. 6.8 ± 21.2 mg/dL, *p* < 0.0001) (Table 3) and FIB-4 (−0.25 ± 0.23 vs. −0.003 ± 0.12, *p* < 0.0001) in the male group than in the female group (Table 3).

## 4. Discussion

Obesity is a multifactorial condition characterized by excessive fat accumulation [1] resulting from complex interactions among physiological, social, cultural, and environmental factors [13]. In 2016, the World Health Organization estimated the global prevalence of obesity at 11% for men and 15% for women, while the World Obesity Federation predicts that by 2030 the prevalence of obesity will be higher in men (one in seven) than in women (one in five) [14].

Epidemiological differences in the prevalence of obesity by sex vary between countries and populations [15]. For example, data from the Italian National Institute of Statistics (ISTAT) in 2021 showed a higher prevalence of being overweight in men compared to women (53.6% vs. 35.7%) [4].

Recognizing the role of sex in health management, funding agencies such as the Canadian Institutes of Health Research, the European Commission, and the U.S. National Institutes of Health have begun to require the inclusion of sex/gender considerations in study design and outcome analyses [6]. However, sex-specific evaluations of anti-obesity drugs remain limited. 

Specifically, among the approved anti-obesity drugs, few studies have examined the sex-specific efficacy of orlistat, with conflicting results [16,17]. Similarly, for naltrexone/bupropion, a higher pharmacokinetic response (e.g., volume of distribution normalized for body weight, maximum plasma concentration) was observed in women than in men, but this was not confirmed by further studies [18,19,20]. 

For liraglutide, few studies have shown sex differences in its effects on weight loss and adverse effects [21], as well as in its pharmacokinetics and pharmacodynamics [6]. Furthermore, although studies have shown that the drug is associated with a 5–10% weight loss rate [22], which is considered clinically relevant for improving obesity-related complications and quality of life [23], it is not clear whether there are sex differences. In addition, limited research has been conducted on the sex response to the beneficial effects of GLP1-RAs on metabolic parameters such as glucose metabolism, lipid profiles, renal function, and hepatic function. This may explain why, to date, there are no published studies suggesting the need for sex-specific dosage recommendations for these drugs [6]. To this end, our study aims to evaluate the sex-specific responses to liraglutide 3.0 mg, the only GLP1-RAs approved in Italy for the treatment of obesity in people without T2D. Given the real-life setting of the study, and according to the inclusion criteria for this pharmacotherapy, we decided to include patients living with obesity (BMI > 30 kg/m^2^). Moreover, in order to evaluate the effects of the drug “per se”, we have excluded the presence of other metabolic comorbidities and their medications.

Our results show a higher prevalence in women (65.9%) than in men (34.0%), which is consistent with electronic medical records from different health care systems and clinical trials of anti-obesity drugs, which have shown that participants with obesity are more likely to be women than men [5,6]. Similarly, Italian data from the international ACTION IO (Awareness Care and Treatment In Obesity Management—an International Observation) study, which assessed the perceptions and barriers to obesity management among people with obesity and healthcare professionals, showed that men perceived weight management as less critical. One reason for this finding appears to be that men believe they can self-control their weight [24]. This observation may explain why a literature review of randomized clinical trials on this topic found difficulties in recruiting men, with an average sex ratio of 27% in men and 73% in women, posing challenges in recruiting participants of the male sex [25].

The main findings of our study are as follows:

1. Both sexes showed significant reductions in WL and BMI after 3 and 6 months of liraglutide treatment, with significantly greater reductions in both weight, and BMI in men. In addition, the percentage of patients achieving WL > 5% at 3 months and WL > 10% at 6 months was significantly higher in men.

As reported in the literature, liraglutide 3.0 mg has demonstrated greater inter-individual variability in weight loss response, but predictive models for individual treatment efficacy have not yet been developed [8]. Studies in the literature suggest that women tend to achieve greater weight loss with all GLP1-RA classes. This was confirmed by a retrospective study showing greater weight loss in women than in men after exenatide treatment (−7.0 kg vs. −3.3 kg) [26], and similar results were observed for BMI, with women showing greater reductions in BMI than men [21,27]. These results are not consistent with our findings, but it must be emphasized that these studies included patients with obesity and concomitant T2D status, different types of GLP1-RAs, and a mean age of patients < 50 years in the latter study.

For liraglutide 3.0 mg, a retrospective analysis of clinical trials showed that women achieved greater weight loss than men, suggesting that men may be less sensitive to liraglutide [7]. However, this analysis included populations with high variability in age, weight, BMI, and drug dose. 

Taken together, the sex-specific effect of liraglutide on weight loss observed in the literature suggests that approximately 50% of the observed sex differences may be due to the greater exposure of women to liraglutide [28], probably related to their lower body surface area associated with their lower baseline body weight [29]. In contrast, another study found that women with higher baseline BMI were associated with greater weight loss [21], but these groups were not homogeneous, as these women were slightly heavier than men at baseline.

Pharmacokinetic analysis has shown that female sex is an independent predictor of increased exposure to liraglutide, which may contribute to improved weight loss outcomes [21]. However, the uniformity of baseline body weight and BMI characteristics between sexes in our study may provide another explanation for these conclusions regarding differential drug exposure. In addition to pharmacokinetic analysis, differences in behavior and attitudes toward medications may also explain how sex influences response to anti-obesity therapies [28].

We hypothesize that possible mechanisms behind the higher response of men than women suggested by our study may be linked to age-related factors. In fact, the mean age of both sexes was about 50 years, which can be considered the perimenopausal period in women, and hormonal changes may affect the results of treatment. In fact, menopause is associated with weight gain and changes in body composition due to increased adipose tissue, especially visceral fat, and decreased lean mass [30].

2. The analysis of metabolic parameters showed a significant reduction in total and LDL cholesterol, and FIB-4 in men compared to women. For HDL cholesterol, no significant differences were observed between men and women at baseline. This may be in contrast to the typically higher levels observed in women. A possible explanation could be that our group of women was in the peri-menopausal period, in which higher abdominal obesity may explain why lower HDL levels were found [31,32].

However, there was no significant sex difference in glucose metabolism and renal function. Previous literature has highlighted sex differences in HbA1c responses to GLP1-RAs, with females showing superiority in the 18–64 age group and males showing superiority in the 65+ age group [21,33]. These differences are difficult to interpret because this study included patients treated with other hypoglycemic agents. In addition, a higher percentage of women than men started with a higher baseline HbA1c (>7%), which was associated with a higher likelihood of achieving glycemic control during follow-up.

Regarding the lipid profile, we observed a greater significant reduction in total cholesterol and LDL cholesterol in men than in women. It is important to evaluate whether the effects of GLP1-RAs on these variables recognized as CVD risk factors show patterns of sexual dimorphism. Although several studies have examined the effect of GLP1-RAs on CVD risk, few have stratified this analysis by sex. Only one study with exenatide showed no reduction in LDL cholesterol in women, suggesting the possible existence of a sex-specific effect of the drug on the lipid profile [21,34]. We hypothesize that these differences could be explained either by direct effects of liraglutide in men or by menopause-related metabolic changes in women, including central adiposity and lipid profile changes [35].

Although the potential reno-protective effects of liraglutide have been documented, sex-specific effects remain underexplored [36]. In our study minimal changes in serum creatinine were observed with no significant sex differences. 

Finally, liraglutide has demonstrated efficacy in improving liver function and reducing the severity of nonalcoholic fatty liver disease (NAFLD) but without sex-based analysis [37,38]. Our results highlighted a reduction in liver enzymes and FIB-4 in both groups but, significantly, slightly higher in men. Since AST and ALT can be normal even in patients with cirrhosis [39], we planned to use FIB-4 as a fibrosis index.

With regard to adverse effects, we reviewed the medical records of all patients to assess whether there were any discontinuations of pharmacotherapy due to side effects. As is known, the most common side effects are gastrointestinal such as nausea and vomiting [21]. In our study, only patients who had reached the maximum dose of liraglutide and who had continued pharmacotherapy for 6 months were included, and no sex differences were observed.

### Limitations and Strengths

Although our study provides valuable information on sex-specific responses to liraglutide therapy, several limitations must be acknowledged. These include the small sample size, which is predominantly female as in the literature, and the retrospective study design. In addition, the study did not account for changes in body composition and possible confounding factors such as dietary habits and physical activity levels. However, in our unit, patients with obesity generally receive counseling regarding both healthy lifestyle and drug titration. We believe that this approach may better explain the drug effect “per se”, independent of the lifestyle effect.

Despite these limitations, the strength of our study lies in its comprehensive real-world evaluation of participants who reached maximum treatment doses after regular titration, thereby improving internal validity. In addition, the focus of our study on a homogeneous cohort of participants goes beyond previous literature and provides further insight into the sex-specific response to liraglutide therapy.

## 5. Conclusions

In conclusion, although the World Health Organization (WHO) declared obesity a global epidemic in 1997 [40], sex/gender-specific considerations are often overlooked in the clinical management of this disease. Our study suggests a sex difference in the response to liraglutide 3.0 mg therapy, with a greater therapeutic effect in men. 

Possible explanations for our findings include sex-specific pharmacodynamics or hormonal influences, which warrant further investigation through large-scale randomized clinical trials to better understand the underlying mechanisms of sex-specific responses.

Given the increased risk of cardiovascular disease (CVD) in men, their reluctance to acknowledge their obesity status [41], and the large impact that improvements in weight, lipid profiles, and liver disease have on CVD risk, our findings may encourage clinicians to increase the involvement of the male population in screening and weight management programs. This proactive approach may be particularly beneficial for men, not only because of their higher cardiovascular risk compared to women but also because they tend to be underrepresented in weight management interventions.

## Figures and Tables

**Table 1:** Baseline (T0) clinical characteristics of the study population (n = 47) treated with liraglutide 3.0 mg.

	Men	Women	*p*-Value
Sex (%)	16/47 (34.0%)	31/47 (65.9%)	
Age (years)	52 ± 14.3	50 ± 11.5	*p* = 0.46
Weight (kg)	115.4 ± 18.1	108.3 ± 12.2	*p* = 0.12
BMI (kg/m^2^)	37.5 ± 5.6	40.1 ± 5.2	*p* = 0.10
FBG (mg/dL)	99.8 ± 20.9	92.3 ± 13.6	*p* = 0.16
HbA1c (%)	5.7 ± 0.54	5.54 ± 0.3	*p* = 0.16
Total cholesterol (mg/dL)	180.2 ± 42.7	180.9 ± 26.6	*p* = 0.95
LDL (mg/dL)	106.0 ± 33.6	103.3± 25.3	*p* = 0.79
HDL (mg/dL)	50.2 ± 6.3	51.3 ± 9.8	*p* = 0.72
TG (mg/dL)	141.6 ± 73.5	115.6 ± 68.0	*p* = 0.31
Creatinine /mg/dL)	1.0 ± 0.2	0.7 ± 0.1	<0.0001
AST (U/L)	23.45 ± 9.4	18.2 ± 4.2	<0.0001
ALT (U/L)	33.2 ± 19.4	20.5 ± 6.7	<0.0001
FIB-4	1.1 ± 0.3	0.7 ± 0.3	<0.0001

Data are mean ± standard deviation (SD) or n (%). BMI: Body Mass Index; HbA1c: glycated hemoglobin; LDL: Low Density Lipoprotein; HDL: High Density Lipoprotein; FBG: blood glucose; TG: triglycerides; FIB-4: Fibrosis-4 index; AST: Aspartate Transaminase; ALT: Alanine Transaminase.

**Table 2:** Changes in weight, BMI, %WL, and %WL > 5–10% after 3 months (T1) and 6 months (T2) of liraglutide treatment.

	Men	Women	*p*-Value
Weight (kg)			
T1–T0	−10.7 ± 6.1	−7.1 ± 3.1	<0.0001
T2–T1	−17.9 ± 6.7	−11.9 ± 5.3	<0.0001
BMI (kg/m^2^)			
T1–T0	−3.6 ± 2.4	−2.6 ± 1.1	*p* = 0.08
T2–T0	−6.0 ± 2.7	−4.4 ± 1.9	*p* = 0.07
%WL			
T1–T0	−9.2 ± 5.1	−6.5 ± 2.9	<0.0001
T2–T1	−15.2 ± 5.4	−10.5 ± 4.4	<0.0001
WL > 5%	N = 15/16, 93.7%	N = 18/31, 58.0%	<0.0001
WL > 10%	N = 14/16, 87.5%	N = 9/31, 29.0%	<0.0001

Data are mean ± standard deviation (SD) or n (%). WL: weight loss; %WL: percentage weight loss; BMI: Body Mass Index.

**Table 3:** Changes in metabolic parameters after 6 months (T2) of liraglutide treatment.

	Men	Women	*p*-Value
FBG (mg/dL) T2–T0	−8.1 ± 16.4	−5.4 ± 9.4	*p* = 0.61
HbA1c (%) T2–T0	−0.23 ± 0.9	−0.16 ± 0.6	*p* = 0.83
Total cholesterol (mg/dL) T2–T0	−14.0± 32.6	9.5 ± 22.1	<0.0001
LDL (mg/dL) T2–T0	−19.0 ± 16.5	6.8 ± 21.2	<0.0001
HDL (mg/dL) T2–T0	−2.0 ± 14.5	1.1 ± 7.1	*p* = 0.14
TG (mg/dL) T2–T0	−9.1 ± 59.9	2.8 ± 51.9	*p* = 0.56
Creatinine (mg/dL) T2–T0	−0.02 ± 0.14	−0.02 ± 0.06	*p* = 0.99
AST (U/L) T2–T0	−3.5 ± 5.6	−2.1 ± 4.8	*p* = 0.54
ALT (U/L) T2–T0	−5.0 ± 15.1	−3.4 ± 7.7	*p* = 0.74
FIB-4 T2–T0	−0.25 ± 0.23	−0.003 ± 0.12	<0.0001

Data are mean ± standard deviation (SD) or n (%). HbA1c: glycated hemoglobin; LDL: Low Density Lipoprotein; HDL: High Density Lipoprotein; FBG: blood glucose; TG: triglycerides; FIB-4: Fibrosis-4 index; AST: Aspartate Transaminase; ALT: Alanine Transaminase.

## Data Availability

The original contributions presented in the study are included in the article, further inquiries can be directed to the corresponding author.
